# Lipids From *Trypanosoma cruzi* Amastigotes of RA and K98 Strains Generate a Pro-inflammatory Response via TLR2/6

**DOI:** 10.3389/fcimb.2018.00151

**Published:** 2018-05-08

**Authors:** Emanuel Bott, Alan B. Carneiro, Guadalupe Gimenez, María G. López, Estela M. Lammel, Georgia C. Atella, Patricia T. Bozza, María L. Belaunzarán

**Affiliations:** ^1^Departamento de Microbiología, Parasitología e Inmunología, Facultad de Medicina, Universidad de Buenos Aires, Buenos Aires, Argentina; ^2^Instituto de Investigaciones en Microbiología y Parasitología Médica, Consejo Nacional de Investigaciones Científicas y Técnicas, Universidad de Buenos Aires, Buenos Aires, Argentina; ^3^Laboratório de Imunofarmacologia, Instituto Oswaldo Cruz, Fundação Oswaldo Cruz, Rio de Janeiro, Brazil; ^4^Instituto Nacional de Tecnología Agropecuaria, Instituto de Biotecnología, Buenos Aires, Argentina; ^5^Consejo Nacional de Investigaciones Científicas y Técnicas, Buenos Aires, Argentina; ^6^Instituto de Bioquímica Médica Leopoldo de Meis, Universidade Federal do Rio de Janeiro, Rio de Janeiro, Brazil

**Keywords:** *Trypanosoma cruzi*, lipids, phospholipase A_1_, pro-inflammatory, TLR2/6

## Abstract

Lipids from microorganisms are ligands of Toll like receptors (TLRs) and modulate the innate immune response. Herein, we analyze *in vitro* the effect of total lipid extracts from *Trypanosoma cruzi* amastigotes of RA and K98 strains (with polar biological behavior) on the induction of the inflammatory response and the involvement of TLRs in this process. We demonstrated that total lipid extracts from both strains induced lipid body formation, cyclooxygenase-2 expression and TNF-α and nitric oxide release in macrophages, as well as NF-κB activation and IL-8 release in HEK cells specifically through a TLR2/6 dependent pathway. We also evaluated the inflammatory response induced by total lipid extracts obtained from lysed parasites that were overnight incubated to allow the action of parasite hydrolytic enzymes, such as Phospholipase A_1_, over endogenous phospholipids. After incubation, these total lipid extracts showed a significantly reduced pro-inflammatory response, which could be attributed to the changes in the content of known bioactive lipid molecules like lysophospholipids and fatty acids, here reported. Moreover, analyses of total fatty acids in each lipid extract were performed by gas chromatography-mass spectrometry. Our results indicate a relevant role of *T. cruzi* lipids in the induction of a pro-inflammatory response through the TLR2/6 pathway that could contribute to the modulation of the immune response and host survival.

## Introduction

*Trypanosoma cruzi* is the etiological agent of Chagas disease that represents a public health problem in Latin America with a recent spreading to non-endemic areas due to human migration; it is currently estimated that nearly 7 million people are infected by this protozoan parasite (WHO Chagas disease (American trypanosomiasis), 2017)^1^. *T. cruzi* is highly heterogeneous in terms of genetics as well as biological behavior and great efforts have been made to identify molecules involved in parasite–host cell interaction, which may also contribute to the pathogenesis of Chagas disease (Zingales et al., 2009). Amastigotes are the replicative stage of this obligate intracellular parasite in the mammalian host, which infect diverse cell types including macrophages, and can form amastigote nests that when degenerate lead to host cell destruction and inflammatory processes with the consequent release of parasite enzymes (Melo and Machado, 2001).

Phospholipids, major components of biomembranes, can be enzymatically modified by the action of phospholipases with generation of bioactive lipids that can act as second messengers and also modulate the immune response. The first evidences related to phospholipid degrading enzymes in *T. cruzi* were associated to the inflammatory responses that appear surrounding degenerating amastigote nests in various tissues of Chagas disease patients, suggesting that the inflammation observed might be attributed to phospholipid breakdown products such as free fatty acids (FFA) and lysophospholipids (Belaunzarán et al., 2011). In addition, we have already demonstrated in all stages of *T. cruzi* RA strain a rapid and extensive breakdown of endogenous phospholipids upon parasite death, with a concomitant accumulation of FFA, that could be attributed to *T. cruzi* Phospholipase A_1_ (Wainszelbaum et al., 2001).

Macrophages initiate the innate immune response by recognizing pathogens through pattern recognition receptors, such as Toll-like receptors (TLRs). Besides, there are growing evidences that point to a modulatory role of lipids from diverse microorganisms in the innate immune response and different members of the TLR family, particularly TLR1, TLR2, TLR4, and TLR6, have been implicated in cell surface recognition of lipids and lipid containing molecules (Roach and Schorey, 2002; Akira and Takeda, 2004; Quesniaux et al., 2004; Gimenez et al., 2010, 2016; Kawai and Akira, 2011). Specifically, TLR2 recognizes a wide variety of pathogen-associated molecular patterns (PAMPs) and forms heterodimers with TLR1 or TLR6 to discriminate between different molecular structures (Akira et al., 2006; Lee et al., 2012). Concerning protozoa, it has been described that parasite molecules from *T. cruzi, T. brucei, Toxoplasma gondii, Leishmania major*, and *Plasmodium falciparum* are sensed by TLRs (Gazzinelli and Denkers, 2006). As regards immune-stimulating molecules of *T. cruzi*, it has been described that trypomastigote mucin glycosylphosphatidylinositol (GPI) anchors are potent stimulators of TLR2/6. In addition, glycoinosiltolphospholipids (GIPLs), free GPI anchors, containing ceramide are recognized by TLR4, while those containing alkylacylglycerol are agonists of TLR2/6 (Cardoso et al., 2016). In the TLRs pathways, several transcription factors including nuclear factor-kappa B (NF-κB) are activated with the consequent induction of inflammatory cytokines, chemokines and nitric oxide (NO) (Akira and Takeda, 2004). In this concern, NF-κB activation is a hallmark of cellular response during *T. cruzi* infection where macrophages have an essential role in the initial control of parasite replication, with the production of inflammatory molecules such as IL-12, IL-8, TNF-α as well as NO that possesses trypanocidal activity (Shoda et al., 2001; Teixeira et al., 2002; Junqueira et al., 2010; Duque and Descoteaux, 2014).

Host-pathogen interaction leads to the formation of lipid bodies (LB) within cells from the immune system. These dynamic organelles, present in the cytoplasm of most eukaryotic cells, are critical regulators of different inflammatory diseases and key markers of leukocyte activation. LB compartmentalize eicosanoid forming enzymes like cyclooxygenase-2 (COX-2), which regulates the production of diverse inflammatory mediators (D'Avila et al., 2008; Bozza et al., 2009). In this regard, it has been reported during *T. cruzi* infection that LB induction in macrophages correlates with the concomitant increase of COX-2 expression (Melo et al., 2003; D'Avila et al., 2011).

In the present work, we investigated the effect of total lipids extracts from *T. cruzi* amastigotes of two strains with polar biological behavior, RA (high virulence) and K98 (low virulence), as well as total lipids extracts obtained from lysed parasites that were overnight incubated (to mimic the enzymatic processes during amastigote nest degeneration where lipids are modified), on the induction of an inflammatory response. Furthermore, lipid modifications were analyzed by thin layer chromatography and total fatty acids by gas chromatography-mass spectrometry. To study the involvement of TLRs in this process, we analyzed NF-κB activation and IL-8 secretion in HEK transfected cells that expressed different combinations of TLRs and were stimulated with the different lipid extracts. Besides, the effect of all these lipids extracts were used to study in murine peritoneal macrophages TNF-α and NO production, LB induction and COX-2 expression.

## Materials and methods

### *T. cruzi* amastigotes

Parasites from two *T. cruzi* strains that belong to different Discrete Typing Units (DTUs): RA (Tc VI, high virulence) and K98 (Tc I, low virulence) were used (González Cappa et al., 1980, 1981; Zingales et al., 2009). To obtain culture amastigotes, J774 E-clone macrophages were infected with bloodstream trypomastigotes from each strain and grown in RPMI 1640 medium (Invitrogen, Grand Island, NY, USA) + 10% fetal bovine serum (Internegocios S.A., Bs. As., Arg.) at 37°C and 5% CO_2_ (Belaunzarán et al., 2013).

### Preparation of total lipid extracts from RA and K98 amastigotes homogenates (RA, K98, RAinc, and K98inc)

Independent batches of amastigotes (1 × 10^9^) of both strains were harvested separately, washed, suspended in phosphate buffer saline (PBS) + 1X protease inhibitor cocktail (Sigma-Aldrich, St. Louis, MO, USA) + 0.1% sodium azide and disrupted by three freezing/thawing cycles (amastigote homogenates). Then, the individual batches from each amastigote homogenate were divided in two (5 × 10^8^ parasites/each): (i) one of them was incubated for 18 h at 37°C, in order to mimic the enzymatic processes that occur during amastigote nest degeneration where parasite lipids are modified (**RAinc** and **K98inc**), and (ii) the other batch was used directly, with no incubation (**RA** and **K98**). In all cases, total lipids were extracted according to Bligh and Dyer (1959), solvents were evaporated with a stream of nitrogen to constant weight and lipids were suspended in ethanol (EtOH) (Merck, Darmstadt, Germany) for cell stimulation assays or in chloroform (Merck) for lipid profile analysis, and stored at −20°C until used.

### Analysis of total lipids from K98 amastigotes

Equal amounts of both **K98** and **K98inc** were separated by thin layer chromatography (TLC) on silica gel 60 plates (Merck) using a double solvent system as previously described (Florin-Christensen et al., 2000). Lipids were identified by comparison with authentic standards. For mass determination, plates were sprayed with 10% CuSO_4_ in 8% H_3_PO_4_ and charred by exposure to 150°C for 13 min (Baron et al., 1984). Densitometric analyses were performed with Gel-Pro® Analyzer 4.0 (Media Cybernetics, Inc., Silver Spring, MD, USA).

### Analyses of fatty acids by gas chromatography-mass spectrometry (GC-MS)

The analyses of the fatty acids (FA) fractions by GC-MS were carried out as previously described (Christie, 2011). Total lipids extracts of parasites (RA, RAinc, K98 and K98inc) were obtained as described above. Each lipid sample was dissolved in 1 ml toluene and 2 ml of 1% sulfuric acid in methanol was added. The mixture was left overnight in a stoppered tube at 50°C, then 1 ml of 5% NaCl was added and the required esters were extracted twice with 2 ml hexane which was removed in a stream of nitrogen. Dried fatty acid methyl esters (FAME) were suspended in 100 μl heptane. GC/MS analyses were carried out on a Shimadzu GCMS-QP2010 Plus system, using an HP Ultra 2 (5% Phenyl-methylpolysiloxane), Agilent (25 m × 0.20 mm × 0.33 μm). Injector was set at 250°C splitless. Column temperature was programmed from 40 to 160°C at 30°C/min, 160–233°C at 1°C/min, 233–300°C at 3°C/min and held at 300°C for 10 min. Helium was used as carrier gas with linear velocity of 36.0 cm s-1. A volume of 2 ml of sample was injected into the chromatograph. Electro ionization (EI-70 eV) and a quadrupole mass analyzer, operated in scans from 40 to 440 amu. Interface was set at 240°C and the ion source at 240°C. The components were identified by comparing their mass spectra with those of the library NIST05 contained in the computer's mass spectrometer. Retention indices were also used to confirm the identity of the peaks in the chromatogram by Supelco 37 Component FAME Mix (Sigma-Aldrich).

### Effect of chlorpromazine on native *T. cruzi* PLA_1_

Aliquots of amastigote homogenates (100 mg/assay) were previously incubated with the *T. cruzi* PLA_1_ inhibitor chlorpromazine (Wainszelbaum et al., 2001) (GlaxoSmithKline Pharmaceuticals, Harlow, UK) at different concentrations (2.5, 5.0, and 10.0 mM), for 30 min, at room temperature and then incubated for 5 h at 37°C. Lipids were extracted and separated by TLC using the double solvent system described above, lipid spots were identified by comparison with commercial standards and mass determination was carried out as described. Densitometric analyses of the lipid spots were performed with Gel-Pro® Analyzer 4.0.

### Effect of recombinant *T. cruzi* PLA_1_ on amastigotes total lipids

#### Cloning and expression of recombinant *T. cruzi* PLA_1_ in a baculovirus expression vector system

The DNA sequence of *T. cruzi* PLA_1_ previously cloned (Belaunzarán et al., 2013), was then cloned into the baculovirus expression vector system using Bac-to-bac methodology according to the supplier's suggestions (Thermo Fisher Scientific Inc., Rockford, IL, USA). Briefly, the plasmid pGEMT-TcPLA_1_ was used as template to amplify the entire open reading frame of *T. cruzi* PLA_1_ gene (GenBank Accession number: JN975637.1) by PCR. using the specific primers 5′ CTC GAG AAC ATG CGC CGC CGC CGC A 3′ and 5′ AAG CTT TCA GTG ATG GTG ATG GTG ATG AGA CTC TCT GTG ACG CGC 3′ which incorporated 5′and 3′ sites for the restriction enzymes XhoI and HindIII, respectively. The 1.029 bp PCR product corresponding to *T. cruzi* PLA_1_ was cloned into pGEM-T easy vector (Promega Corporation, Madison, WI, USA) and sub-cloned into pFastBac1 (pFB1) donor vector (Thermo Fisher Scientific Inc.) using Xho I and Hind III, under the control of polyhedrin promoter. The transfer vectors (pFB1-*T. cruzi* PLA_1_) were used to transform DH10Bac cells (Thermo Fisher Scientific Inc.,). White colonies were selected by addition of kanamycin, tetracycline and gentamycin in the presence of IPTG and Bluo-Gal.The presence of the transgene and absence of not-recombinant DNA was confirmed by PCR using M13 forward and M13 reverse primers. The correct recombinant bacmid DNA was transfected to Sf9 cells derived from the fall armyworm *Spodoptera frugiperda*, using Cellfectin II transfection reagent, according to the manufacturer's instructions (Thermo Fisher Scientific Inc.,). Transfection supernatants were collected after 5 days and the viral stocks were tittered by end point dilution (O'Reilly et al., 1994).

For expression of recombinant *T. cruzi* PLA_1_, Sf9 cells were infected with high-titer virus stock previously obtained and harvested at 48–96 h post-infection. For *T. cruzi* PLA_1_ purification, cells were washed twice with PBS, suspended in lysis buffer (50 mM NaH_2_PO_4_, 300 mM NaCl, 10 mM Imidazole, pH 7.6) in the presence of 1X protease cocktail inhibitor and incubated at 4°C for 30 min. The lysate was centrifuged at 10,000 × g for 20 min at 4°C and the clear supernatant was collected and filtered through 0.45 μm membrane and then loaded onto a nickel resin column (Thermo Fisher Scientific Inc.). Recombinant *T. cruzi* PLA_1_ was eluted by pH gradient using 50 mM NaH_2_PO_4_, 300 mM NaCl, pH 4 to 5. Aliquots were analyzed by SDS-PAGE, Comassie blue staining, immunoblot and phospholipase activity (Reisfeld et al., 1994; Belaunzarán et al., 2013).

#### Analysis of amastigote lipid degradation by recombinant *T. cruzi* PLA_1_

Amastigotes (1 × 10^9^), RA strain, were washed thrice in PBS, suspended in 10 mM Tris with protease inhibitors (1X protease cocktail inhibitor; 0.5 mM TLCK, 2.5 mM E-64) and disrupted by five cycles of freezing and thawing. Total lipids were extracted as described (Bligh and Dyer, 1959), solvents were evaporated with a stream of nitrogen to constant weight and lipids were then suspended in 0.2 M sodium acetate, pH 4.7. Then, 500 μl of total lipid suspensions (5 mg/ml) were incubated with 200 μl of recombinant *T. cruzi* PLA_1_ (700 μg/ml) or 200 μl of elution buffer as control, at 37°C for 18 h. Lipids were extracted and separated by TLC using a double solvent system and identified by comparison with commercial standards. Mass determination and densitometric analyses of the lipid spots were performed as described above.

### Human embryonic kidney 293A cells adherent clone (HEK 293A cells)

HEK 293A cells were cultured in high-glucose Dulbecco's modified Eagle medium supplemented with 10% fetal bovine serum (FBS) in the absence of antibiotics, at 37°C and 5% CO_2_. These cells were used to evaluate TLRs participation in NF-κB activation and IL-8 release triggered by the different lipid extracts.

### TLRs transfection assay of HEK 293A cells

TLRs transfection of HEK 293A cells was performed as previously described (Carneiro et al., 2013). Briefly, 5 × 10^5^cells/well were seeded into 12-well plates for 24 h at 37°C, 5% CO_2_ and then transfected using Lipofectamine 2000 and Opti-MEM (Invitrogen) according to manufacturer's instructions. To analyze TLR2/6, TLR2/1 and TLR4 involvement, the following combinations of plasmid constructs were used: (A) TLR2, TLR6, CD14, CD36, MD-2, Firefly luciferase reporter construct driven by a NF-κB-dependent promoter (Firefly), β-actin-Renilla luciferase reporter construct used as transfection efficiency control (Renilla) and pDisplay (Invitrogen); (B) TLR2, TLR1, CD14, CD36, MD-2, Firefly, Renilla and pDisplay; (C) TLR4, CD14, CD36, MD-2, Firefly, Renilla and pDisplay; and (D) Firefly, Renilla and pDisplay (Empty vector). The amounts of constructs per well were: 0.20 μg mouse TLR2 or TLR4 or TLR6, 0.80 μg mouse TLR1, 0.20 μg mouse MD-2, CD14 and CD36, 0.20 μg Firefly, 6.60 ng Renilla. To reach a constant DNA mass of 2.00 μg, different amounts of the pDisplay were then added in each well. Cells were grown for 24 h, detached by trypsin treatment and incubated for 24 h in 96-well plates (4 × 10^4^/well) at 37°C, 5% CO_2_.

### NF-κB activation assessment by luciferase reporter assay

HEK 293A transfected cells were stimulated for 4 h at 37°C, 5% CO_2_, with 0.5, 5, or 50 μg/ml of **RA**, **RAinc**, **K98**, and **K98inc** or 100 ng/ml lipopolysaccharide (LPS, TLR4 ligand) or 1 nM Pam3CSK4 (P3C, TLR2/1 ligand) or 10 ng/ml fibroblast-stimulating lipopeptide-1 (FSL-1, TLR2/6 ligand) (InvivoGen, San Diego, CA, USA) or 0.5% EtOH (vehicle) as control. Cells were then washed with PBS, lysed in passive lysis buffer (Promega, Madison, WI, USA) and NF-κB activation was evaluated through luciferase activity determination using the Dual-Luciferase Reporter Assay System, according to the manufacturer's instructions (Promega). Relative luminescence units were determined with the SpectraMax luminometer (Thermo Scientific, Meridian Rd, Rockford, IL, USA) and luciferase activity was expressed as Firefly/Renilla luciferase activity ratio.

### IL-8 chemokine determination

HEK 293A transfected cells were stimulated for 20 h with 0.5, 5, or 50 μg/ml of **RA**, **RAinc**, **K98**, and **K98inc** or 100 ng/ml LPS or 1 nM P3C or 10 ng/ml FSL-1 or 0.5% EtOH (vehicle) as control. IL-8 levels were determined in cell supernatants by ELISA using Human CXCL8/IL-8 DuoSet ELISA Kit (R&D Systems, Minneapolis, MN, USA).

### Murine peritoneal macrophages

Peritoneal macrophages were obtained from BALB/c mice as previously described (Gimenez et al., 2010) and cultured in RPMI 1640 medium supplemented with 100 U/ml penicillin and 100 μg/ml streptomycin at 37°C, 5% CO_2_. Local guidance for animal care and experimentation were followed throughout this research according to protocols approved by the Universidad de Buenos Aires's Institutional Committee for the Care and Use of Laboratory Animals (CICUAL) in accordance with the Council for International Organizations of Medical Sciences (CIOMS) and International Council for Laboratory Animal Science (ICLAS) international ethical guidelines for biomedical research involving animals.

### Determination of lipid bodies (LB)

Macrophages (1 × 10^6^/well) were plated onto coverslips in 24-well plates and stimulated with 50 μg/ml of the different lipid extracts or 0.5% EtOH (vehicle) as negative control or 100 ng/ml LPS as positive control for 24 h at 37°C, 5% CO_2_. For LB quantification, cells were fixed in 4% formaldehyde while still moist, washed and stained with 0.5% Oil red O (Melo et al., 2011). Cell morphology was visualized and LB were counted in 50 consecutive macrophages using phase contrast microscopy (100x, Nikon Eclipse E600).

### COX-2 expression

For COX-2 immunoblot analysis, 1 × 10^6^ macrophages/well were plated in 24-well plates and stimulated with 50 μg/ml of the different lipid extracts or 0.5% EtOH (vehicle) as negative control or 100 ng/ml LPS as positive control for 24 h at 37°C, 5% CO_2_. Cells were then washed with PBS, homogenized with lysis buffer (50 mM NaH_2_PO_4_, 300 mM NaCl, pH 8) and suspended in Laemmli sample buffer + 100 mM DTT, boiled and stored at −20°C until used. Immunoblot analyses were performed as previously reported using goat IgG anti COX-2 (1:200 v/v, Santa Cruz Biotechnology, CA, USA), and anti-goat IgG-HRP conjugate (1:2000 v/v, Santa Cruz Biotechnology, TX, USA) (Gimenez et al., 2016). For loading control, detection of β-actin was performed in the same membranes. The COX-2 band intensity was quantified by densitometry using Gel-Pro® Analyzer 4.0 and normalized to that of the corresponding β-actin band.

### Nitric oxide determination

Macrophages (5 × 10^5^/well) were plated in 96-well plates and stimulated with 50 μg/ml of the different lipid extracts or 0.5% EtOH (vehicle) as negative control or 100 ng/ml LPS as positive control, in the presence of 600 pg/ml IFN-γ, for 48 h at 37°C, 5% CO_2_. NO levels were determined in cell supernatants as nitrite production using the Griess reaction and absorbance at 540 nm (Gimenez et al., 2013).

### TNF-α measurement

Macrophages (1 × 10^6^/well) were plated in 24-well plates and stimulated with 50 μg/ml of the different lipid extracts or 0.5% EtOH (vehicle) as negative control, or 100 ng/ml LPS as positive control, for 24 h at 37°C, 5% CO_2_. TNF-α levels were determined by ELISA in cell supernatants according to manufacturer's protocol (R&D Systems, Minneapolis, MN, USA).

### Statistical analysis

The results were expressed as mean ± SEM. Data were analyzed by one-way analysis of variance (ANOVA) followed by Bonferroni's Multiple Comparison Test using GraphPad Prism 4.0. Statistically significant differences were represented as ^*^p < 0.05; ^**^p < 0.01 and ^***^p < 0.001.

## Results

### *T. cruzi* total lipid extracts possess quantitative differences in their lipid profiles

**Figure 1:**
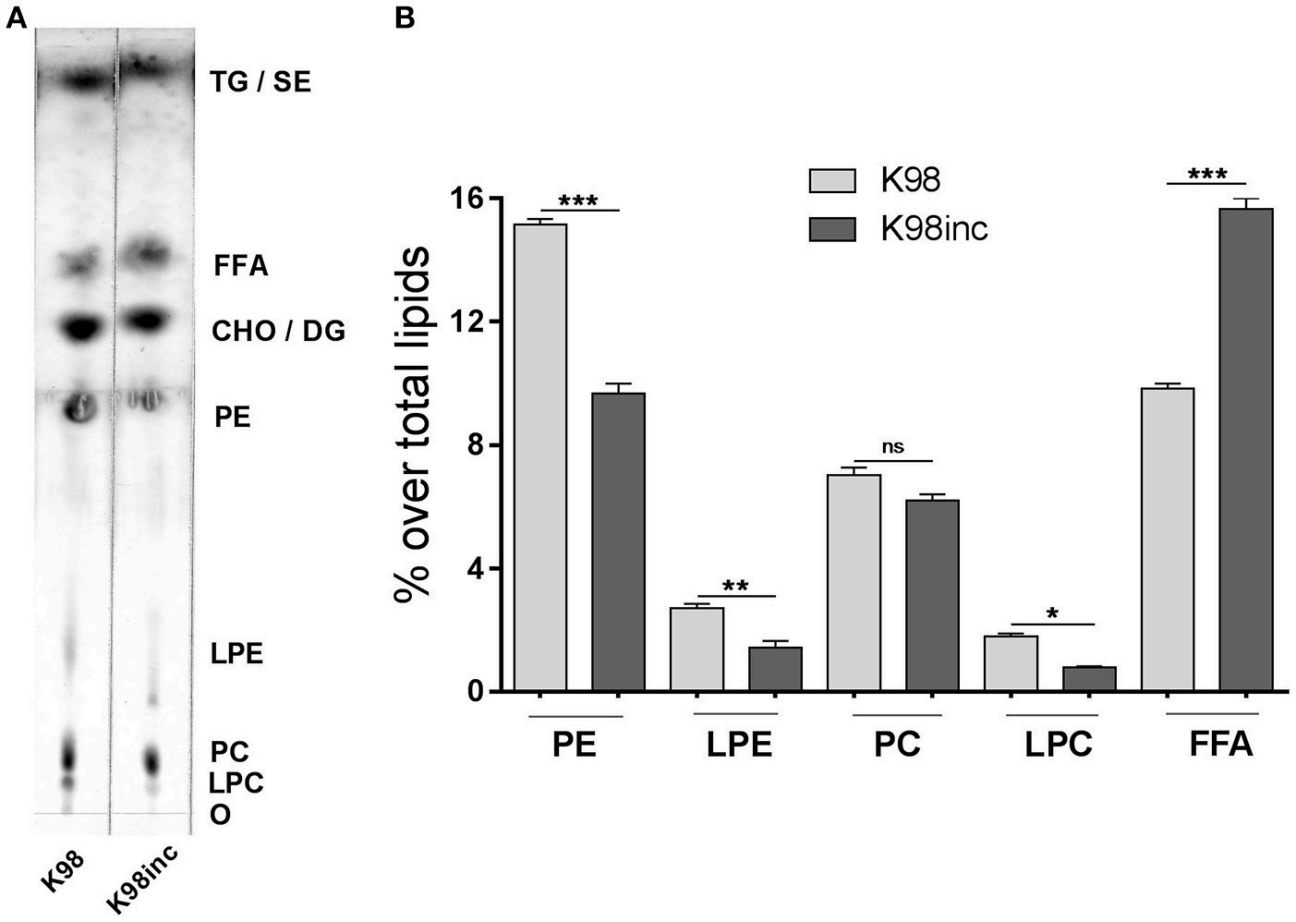
Analysis of the lipid composition of K98 and K98inc. **(A)** K98 and K98inc were separated by TLC using a double solvent system. For mass determination, plates were sprayed with 10% CuSO_4_ in 8% H_3_PO_4_ and charred by exposure to 150°C for 13 min; lipids were identified by comparison with authentic standards. The TLC plate is representative of three independent experiments performed with independent batches of lipid extracts. **(B)** Densitometric analyses of the TLC plate were performed and the percentage of each lipid fraction was determined with respect to total lipids for each lane. Each bar represents the mean ± SEM of triplicate determinations. O, origin; LPC, Lysophosphatidylcholine; PC, Phosphatidylcholine; LPE, Lysophosphatidylethanolamine; PE, Phosphatidylethanolamine; CHO, Cholesterol; DG, Diacylglycerol; FFA, Free fatty acids; TG, Triacylglycerol; SE, Steryl esters; K98, total lipid extract from amastigotes of K98 *T. cruzi* strain; K98inc, total lipid extract from incubated amastigotes of K98 *T. cruzi* strain; ^*^, ^**^ and ^***^ statistically significant (*p* < 0.05, *p* < 0.01, and *p* < 0.001 respectively).

Previously, we reported differences in the profiles of total lipid extracts from *T. cruzi* amastigotes of the highly virulent RA strain (**RA**) with respect to those from parasites incubated for 12 h at 37°C (**RAinc**). In RA the main phospholipid was phosphatidylcholine (PC) which was degraded when parasites were incubated, observing a simultaneous and significant increase in FFA generation, facts that could be attributed to parasite phospholipase and lysophospholipase degrading activities (Wainszelbaum et al., 2001). Herein, we analyzed by TLC the total lipid extract from *T. cruzi* amastigotes of the low virulence strain K98 (**K98**) as well as that corresponding to incubated parasites (**K98inc**). Figure 1A shows that K98 and K98inc possess quantitative differences in their lipid profiles, being phosphatidylethanolamine (PE) the main phospholipid in both of them. The densitometric analyses indicated a significant decrease in PE, lysophosphatidylethanolamine (LPE) and lysophosphatidylcholine (LPC) (~36, 48, and 56% respectively) with a concomitant increase in FFA (~ 59%) in K98inc with respect to K98 (Figure 1B).

**Figure 2:**
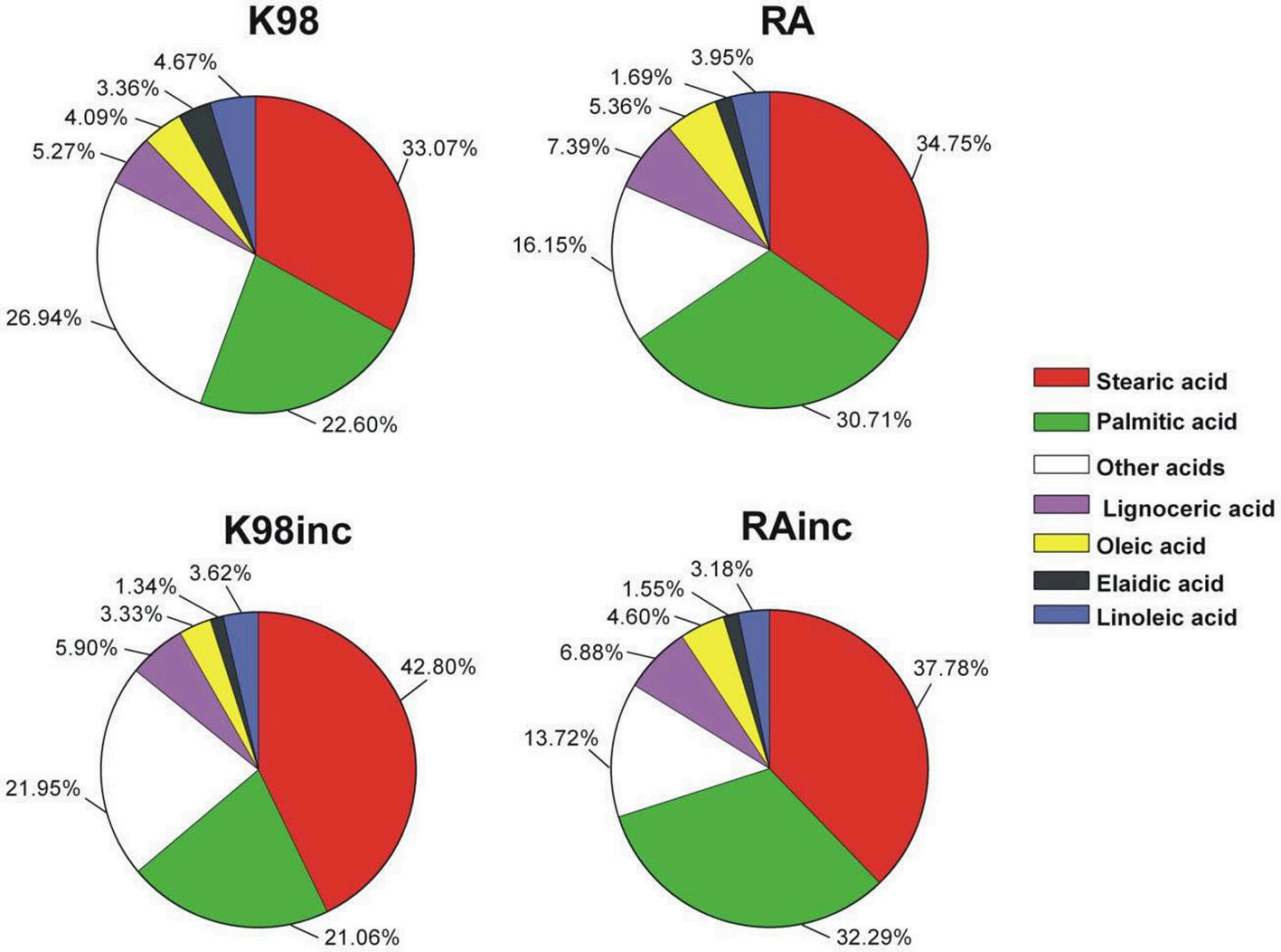
The most abundant fatty acids present in the different *T. cruzi* amastigote lipid extracts were determined by Gas Chromatography–Mass Spectrometry (GC-MS) analyses and are displayed in cake graph format. RA: total lipid extract from amastigotes of RA *T. cruzi* strain; RAinc: total lipid extract from incubated amastigotes of RA *T. cruzi* strain; K98: total lipid extract from amastigotes of K98 *T. cruzi* strain; K98inc: total lipid extract from incubated amastigotes of K98 *T. cruzi* strain.

To determine the identity of the fatty acids (FA) present in the different *T. cruzi* extracts, we performed a GC–MS analysis (Table 1). The most abundant FAs for all the extracts were stearic acid and palmitic acid followed by lignoceric acid (Figure 2). As regards RA vs K98, we determined differences in the relative amounts of the main FA: RA presented higher levels of palmitic acid, lignoceric acid and oleic acid with respect to K98 (~36, 40, and 31% higher, respectively), whereas 4,7,10,13,16,19-docosahexaenoic acid, elaidic acid, linoleic acid, arachidonic acid and myristic acid were present in higher proportions in K98 than RA (~72, 99, 18, 76, and 24% higher, respectively). Considering K98inc vs. K98, we detected a ~29% increase in stearic acid in the former, whereas RAinc vs RA displayed a ~9% increase in stearic acid and 5% in palmitic acid (Table 1).

**Table 1:** Fatty acids analyses of RA, RAinc, K98 and K98inc. The analyses of the fatty acids (FA) fractions of all *T. cruzi* total lipid extracts were performed by GC–MS.

**Compounds**	**% over total FA**			
	**K98**	**K98inc**	**RA**	**RAinc**
Lauric acid	1.02	0.00	0.37	0.17
Myristic acid	2.24	0.37	1.81	1.36
Pentadecanoic acid	0.47	0.15	0.63	0.34
Palmitoleic acid	0.23	0.16	0.43	0.36
Palmitic acid	22.59	21.06	30.71	32.29
Margaric acid	0.66	0.50	0.47	0.47
Linoleic acid	4.67	3.62	3.95	3.18
Oleic acid	4.09	3.33	5.36	4.60
Elaidic acid	3.36	1.34	1.69	1.55
Stearic acid	33.07	42.79	34.75	37.78
Arachidonic acid	1.85	1.64	1.05	0.90
8,11,14-Eicosatrienoic acid	0.42	0.38	0.29	0.27
11,14-Eicosadienoic acid	0.21	0.00	0.00	0.00
Arachidic acid	0.47	0.31	0.20	0.27
4,7,10,13,16-Docosapentaenoic acid	1.87	1.57	1.41	1.14
4,7,10,13,16,19-Docosahexaenoic acid	3.74	3.20	2.17	1.76
7,10,13,16-Docosatetraenoic acid	1.41	1.26	0.53	0.45
7,10,13,16,19-Docosapentaenoic acid	1.31	1.21	0.62	0.52
Erucic acid	0.37	0.30	0.26	0.23
Behenic acid	0.84	0.57	0.48	0.41
Tricosanoic acid	0.20	0.19	0.13	0.15
Nervonic acid	0.77	0.83	0.42	0.38
Lignoceric acid	5.27	5.90	7.39	6.88
Cerotic acid	0.58	0.47	0.80	0.70
Cholesterol	8.29	8.83	4.08	3.86

*The components were identified by comparing their mass spectra with those of the library NIST05 contained in the computer's mass spectrometer. The FA are listed in ascending order according to the retention time and their content is presented as the percentage of each FA with respect to the total FA present in each sample*.

### Amastigote phospholipids are hydrolyzed by *T. cruzi* PLA_1_

**Figure 3:**
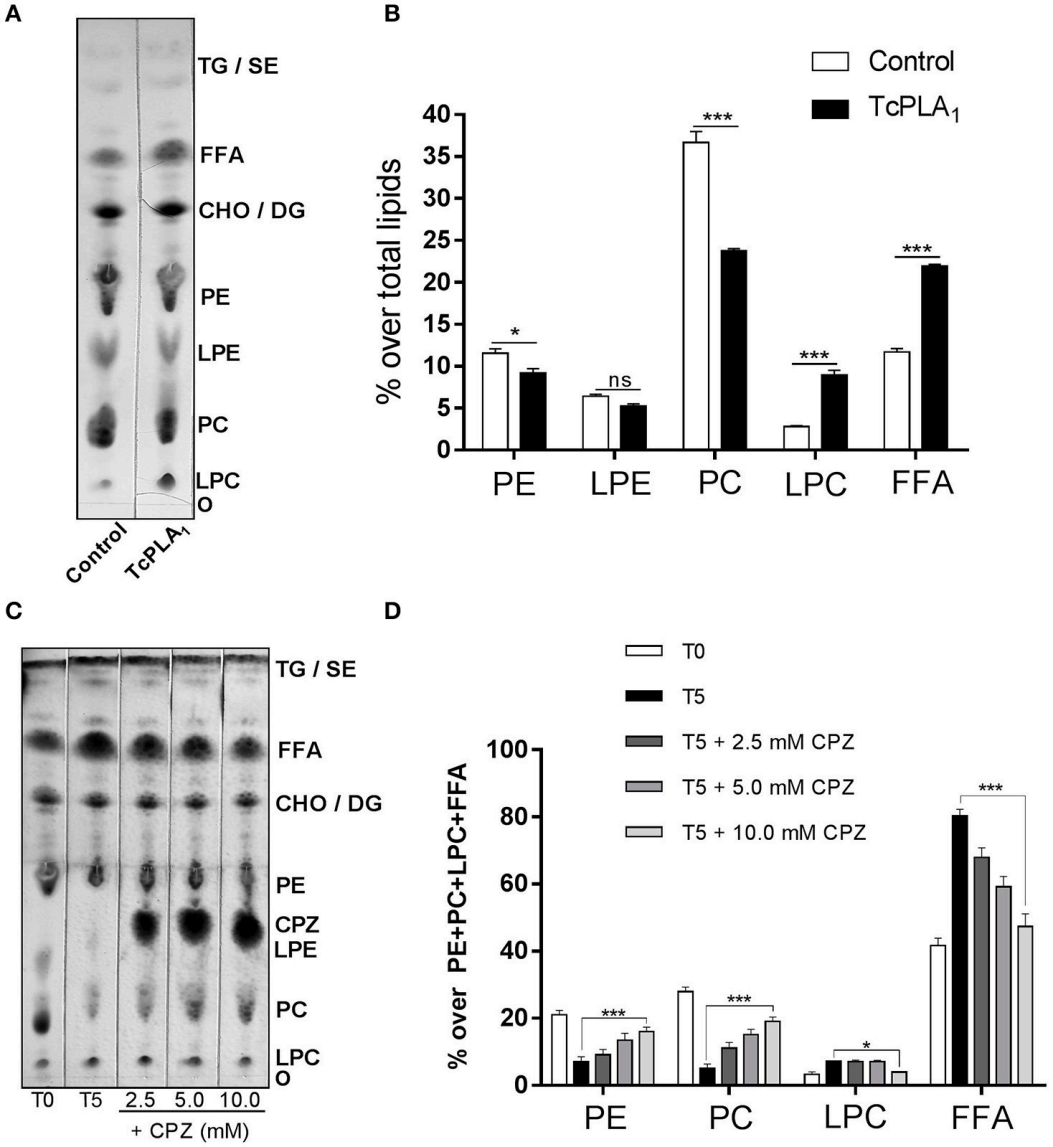
Amastigote phospholipids are hydrolyzed by *T. cruzi* PLA_1_. *Effect of recombinant T. cruzi PLA*_1_ *on amastigotes total lipids*. **(A)** Amastigotes total lipid suspensions from *T. cruzi* RA strain (5 mg/ml) were incubated with 700 μg/ml recombinant *T. cruzi* PLA_1_ or elution buffer as control, at 37°C for 18 h. Lipids were extracted and separated by TLC and identified by comparison with commercial standards. The TLC plate is representative of two independent experiments performed with independent batches of lipid extracts. Mass determination was carried out by charring. **(B)** Densitometric analyses of the TLC plate were performed with Gel-Pro® Analyzer 4.0 and the percentage of each lipid fraction was determined with respect to total lipids for each lane. Each bar represents the mean ± SEM of triplicate determinations. **(C)** *Effect of chlorpromazine on native T. cruzi PLA*_1_. Amastigote homogenates were pre-incubated with the *T. cruzi* PLA_1_ inhibitor chlorpromazine (CPZ) at different concentrations (2.5, 5.0, and 10.0 mM) at room temperature and then incubated for 5 h at 37°C. Lipids were then extracted and separated by TLC, lipid spots identified by comparison with commercial standards and mass determination was carried out by charring. As controls, lipids from amastigote homogenates without CPZ, non-incubated (T0) and 5 h incubated (T5), were included. **(D)** Densitometric analyses of the TLC plates were performed with Gel-Pro® Analyzer 4.0 and the percentage of each lipid fraction was determined over PE + PC + LPC + FFA content for each lane. Each bar represents the mean ± SEM of triplicate determinations of 2 independent assays. O, origin; LPC, Lysophosphatidylcholine; PC, Phosphatidylcholine; LPE, Lysophosphatidylethanolamine; PE, Phosphatidylethanolamine; CHO, Cholesterol; DG, Diacylglycerol; FFA, Free fatty acids; TG, Triacylglycerol; SE, Steryl esters; TcPLA_1_, *Trypanosoma cruzi* Phospholipase A_1_; ^*^ and ^***^ statistically significant (*p* < 0.05 and *p* < 0.001).

To demonstrate if *T. cruzi* PLA_1_ participates in the degradation of endogenous phospholipids observed after homogenates incubation, two different experimental approaches were performed. First, we evaluated the effect of recombinant *T. cruzi* PLA_1_ on amastigote total lipids. For this purpose, this enzyme was subcloned into a baculovirus expression system (Supplementary Figure 1) and then purified (Supplementary Figure 2); lipids were extracted from amastigote homogenates in order to remove native *T. cruzi* PLA_1_ which could hydrolyze endogenous phospholipids, and finally, lipids were incubated with recombinant *T. cruzi* PLA_1_. Figure 3A shows that this enzyme significantly hydrolyzed parasite lipids and the densitometric analyses displayed a significant decrease in PC and PE (~35 and 20%, respectively) with a concomitant increase in LPC and FFA (~218 and 88%, respectively), with respect to control (Figure 3B). Second, we investigated whether pre-treatment of amastigote homogenates with chlorpromazine, a *T. cruzi* PLA_1_ inhibitor (Wainszelbaum et al., 2001), could abrogate the lipid modifications previously determined (Figure 1). Figure 3C shows that this compound significantly inhibited PC and PE degradation as well as FFA and LPC generation, in a dose dependent manner. Considering that LPE and chlorpromazine presented a similar retention factor, we were not able to analyze and discriminate any variations in this spot. Densitometric analyses showed that the variations observed in PC, PE, LPC, and FFA were significant with respect to control (Figure 3D).

### RA and K98 triggered the highest NF-κB activation and IL-8 secretion via TLR2/6

**Figure 4:**
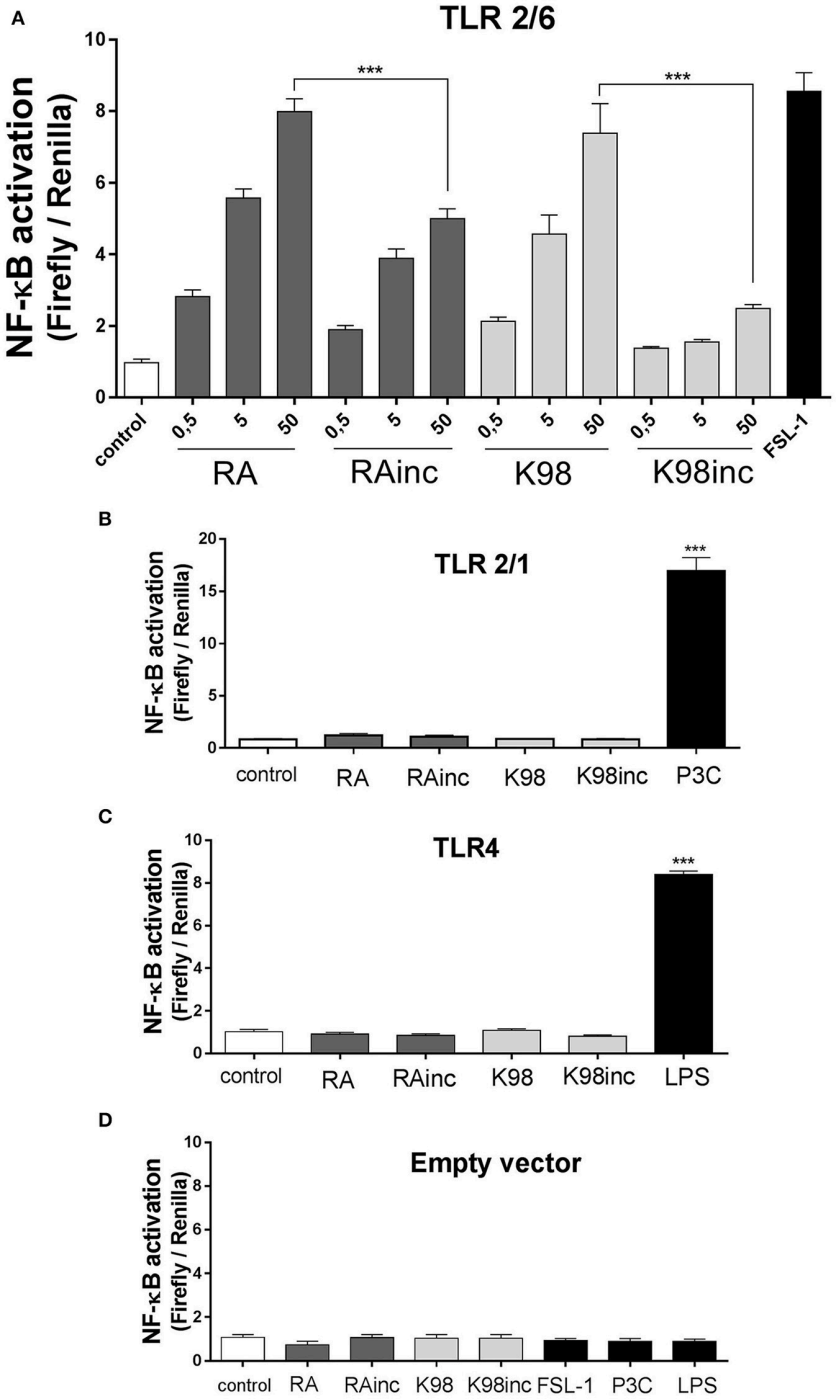
RA and K98 triggered the highest NF-κB activation via TLR2/6. HEK 293A cells were transfected with four different combinations of plasmid constructs: **(A)** TLR2, TLR6, CD14, CD36, MD-2, Firefly luciferase reporter construct driven by a NF-κB -dependent promoter (Firefly), β-actin-Renilla luciferase control reporter (Renilla), and pDisplay; **(B)** TLR2, TLR1, CD14, CD36, MD-2, Firefly, Renilla, and pDisplay; **(C)** TLR4, CD14, CD36, MD-2, Firefly, Renilla, and pDisplay; and **(D)** Firefly, Renilla, and pDisplay (empty vector). Cells were then stimulated for 4 h with 0.5, 5, or 50 μg/ml of RA, RAinc, K98 and K98inc or 100 ng/ml LPS or 1 nM P3C or 10 ng/ml FSL-1 or 0.5% EtOH (control). NF-κB activation was evaluated through luciferase activity determination and expressed as Firefly/Renilla ratio. **(B–D)** show the results of cells stimulated with the highest concentration of each lipid extract (50 μg/ml). Each bar represents the mean ± SEM of triplicate determinations from three independent experiments. FSL-1, fibroblast-stimulating lipopeptide-1; LPS, lipopolysaccharide; P3C, Pam3CSK4; RA, total lipid extract from amastigotes of RA *T. cruzi* strain; RAinc, total lipid extract from incubated amastigotes of RA *T. cruzi* strain; K98: total lipid extract from amastigotes of K98 *T. cruzi* strain; K98inc: total lipid extract from incubated amastigotes of K98 *T. cruzi* strain; ^***^ statistically significant (*p* < 0.001).

We next analyzed if RA, K98, RAinc and K98inc were able to induce NF-κB activation and IL-8 secretion as well as which of the TLRs combinations in HEK transfected cells (TLR2/6, TLR2/1, or TLR4), were involved in these processes. Figure 4A shows that all lipid extracts induced NF-κB activation in HEK cells through the heterodimeric complex TLR2/6 and in a dose-dependent manner. In particular, RA and K98 generated significantly higher levels of activation with respect to RAinc and K98inc (~1.60 and 2.98-fold higher, respectively). In contrast, no differences in NF-κB activation were observed respect to control when TLR2/1 or TLR4 transfected cells were stimulated with each lipid extract even at the higher dose, whereas treatments with P3C or LPS were able to activate NF-κB, thus confirming cell responsiveness (Figures 4B,C).

**Figure 5:**
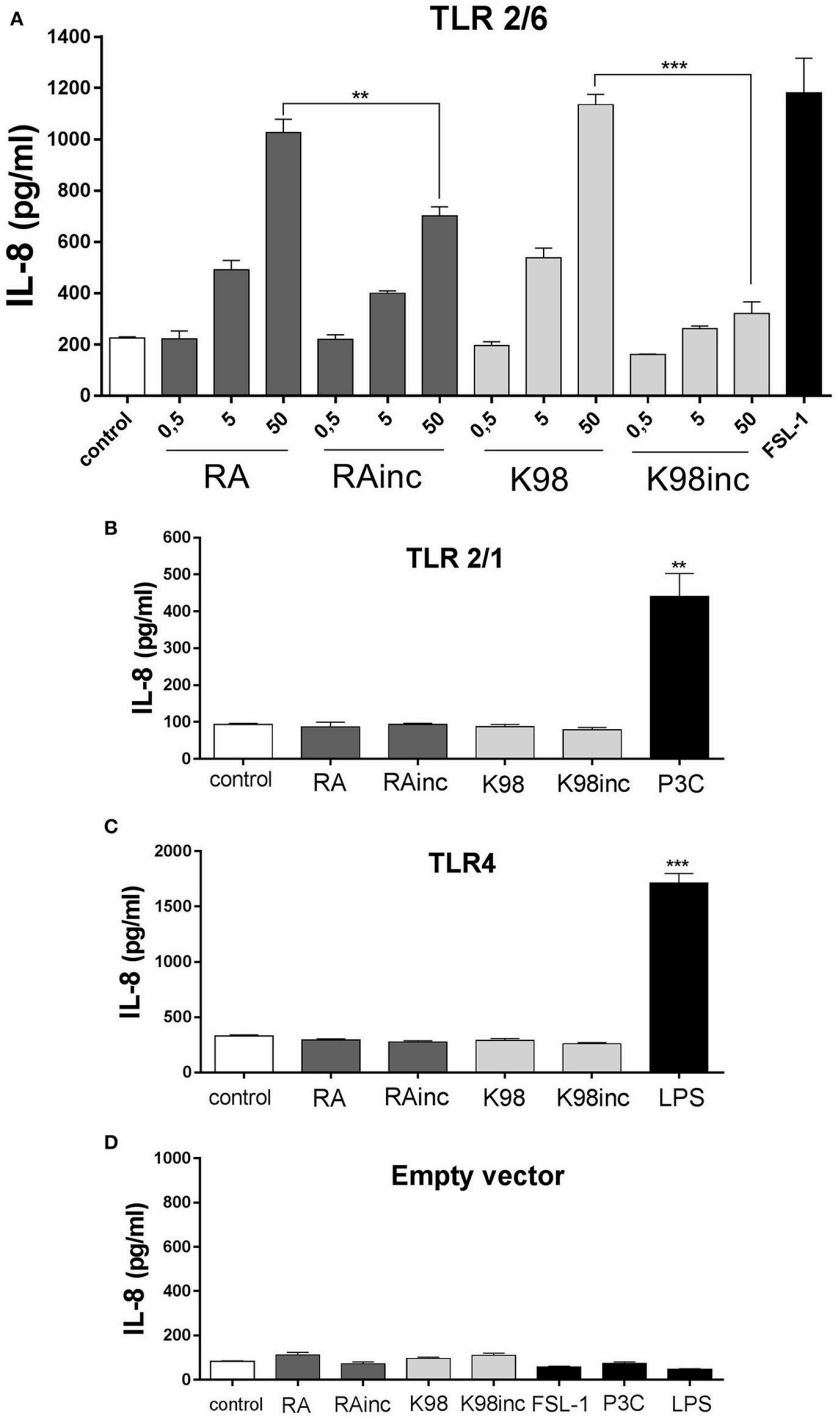
RA and K98 triggered the highest IL-8 secretion via TLR2/6. HEK 293A cells were transfected with four different combinations of plasmid constructs: **(A)** TLR2, TLR6, CD14, CD36, MD-2, Firefly luciferase reporter construct driven by a NF-κB -dependent promoter (Firefly), b-actin-Renilla luciferase control reporter (Renilla), and pDisplay; **(B)** TLR2, TLR1, CD14, CD36, MD-2, Firefly, Renilla, and pDisplay; **(C)** TLR4, CD14, CD36, MD-2, Firefly, Renilla, and pDisplay; and **(D)** Firefly, Renilla, and pDisplay (empty vector). Cells were then stimulated with 0.5, 5, or 50μg/ml of RA, RAinc, K98 and K98inc or 100 ng/ml LPS or 1 nM P3C or 10 ng/ml FSL-1 or 0.5% EtOH (control). After 20 h of incubation, IL-8 levels were determined in the supernatants by ELISA. Each bar represents the mean ± SEM of triplicate determinations from three independent experiments; FSL-1, fibroblast-stimulating lipopeptide-1; LPS, lipopolysaccharide; P3C: Pam3CSK4; RA, total lipid extract from amastigotes of RA *T. cruzi* strain; RAinc: total lipid extract from incubated amastigotes of RA *T. cruzi* strain; K98, total lipid extract from amastigotes of K98 *T. cruzi* strain; K98inc, total lipid extract from incubated amastigotes of K98 *T. cruzi* strain; ^**^ and ^***^ statistically significant (*p* < 0.01 and *p* < 0.001 respectively).

Concerning IL-8, similar results to that determined for NF-κB activation were obtained, as all lipid extracts were able to induce this chemokine release also through TLR2/6 and in a dose-dependent manner. Once again, RA and K98 generated significantly higher levels of IL-8 release with respect to RAinc and K98inc (~1.40 and 3.50-fold higher, respectively) (Figure 5A) and neither TLR2/1 nor TLR4 were involved in lipid recognition (Figures 5B,C). Noteworthy, no differences in NF-κB activation and IL-8 release were observed in cells transfected with the empty vector and stimulated with the same lipid extracts or classical TLR ligands with respect to control, indicating that all the results here obtained were dependent on TLR expression and not mediated by other receptors present in HEK cells (Figures 4D, 5D).

### RA and K98 induced LB formation and COX-2 expression

**Figure 6:**
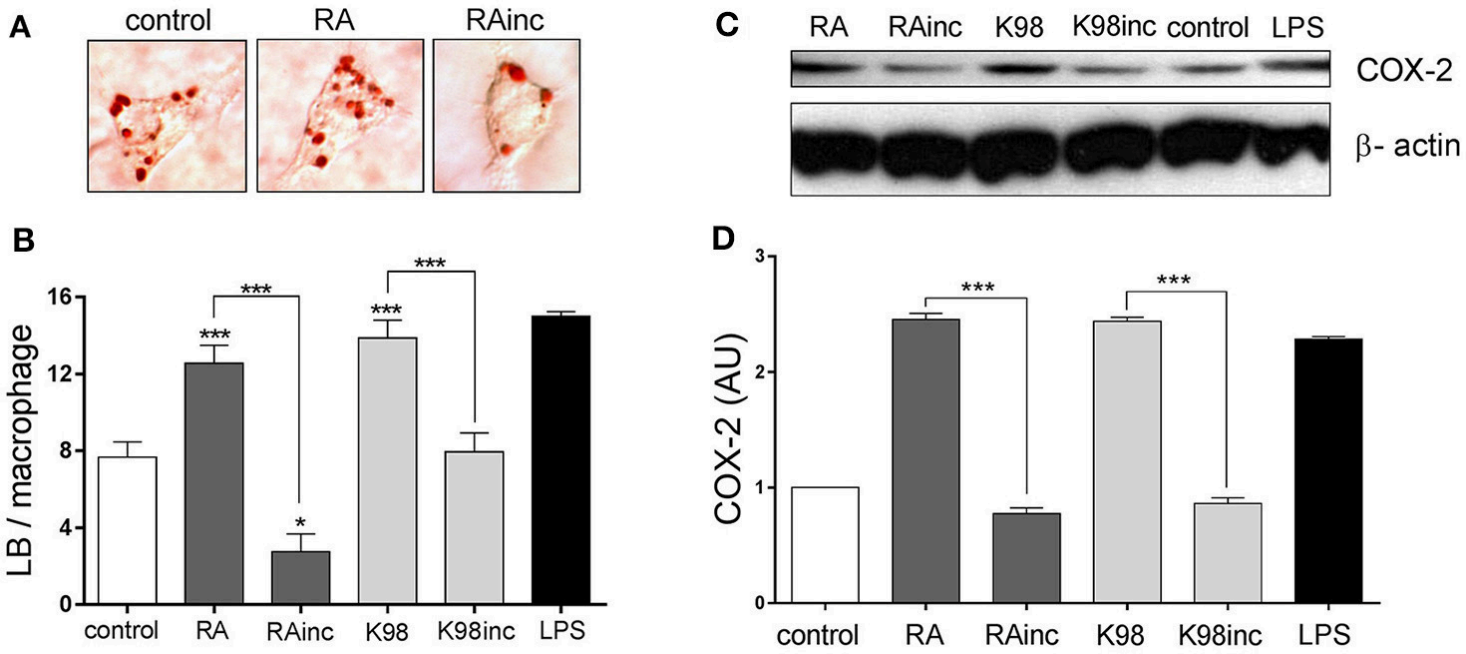
RA and K98 induced the formation of lipid bodies (LB) and COX-2 expression. Macrophages were stimulated for 24 h with 50 μg/ml of RA, RAinc, K98 or K98inc; 0.5% EtOH (control) or 300 ng/ml LPS and then staining with Oil red O. **(A)** Lipid bodies in macrophages stimulated with RA or RAinc and control cells. **(B)** Lipid bodies were counted in 50 consecutive macrophages and each bar represents the mean ± SEM from three independent experiments. **(C)** Immunoblot analysis of COX-2 expression and β-actin (loading control) are representative of three independent experiments. **(D)** The intensity of COX-2 bands was quantified by densitometry and normalized to the intensity to the corresponding β-actin band. Data represent the mean ± SEM of triplicate determinations. RA, total lipid extract from amastigotes of RA *T. cruzi* strain; RAinc, total lipid extract from incubated amastigotes of RA *T. cruzi* strain; K98, total lipid extract from amastigotes of K98 *T. cruzi* strain; K98inc, total lipid extract from incubated amastigotes of K98 *T. cruzi* strain, AU, arbitrary units; ^*^ and ^***^ statistically significant (*p* < 0.05 and *p* < 0.001).

It is known that *T. cruzi* infection induces in macrophages LB formation, structural markers of inflammation (Melo and Dvorak, 2012). We here demonstrated that both RA and K98 were able to induce LB formation in murine peritoneal macrophages with respect to control (Figure 6A). In the case of RAinc, LB generation was significantly inhibited with respect to control and when compared with RA, the reduction in the number of LB was ~4.57 folds (Figures 6A,B). K98inc had no effect with respect to control and we observed a reduction in LB numbers of ~1.74-fold lower with respect to K98 (Figure 6B). Considering that LB contain eicosanoid-forming enzymes such as COX-2, we then investigated if the lipid extracts were able to induce this enzyme expression in murine peritoneal macrophages. Results show that both RA and K98 induced COX-2 expression with respect to control, whereas RAinc and K98inc did not. When comparing RAinc vs RA and K98inc vs K98, we observed a reduction in COX-2 levels of ~3.18 and 2.84 folds respectively (Figures 6C,D).

### RA and K98 stimulated NO production

**Figure 7:**
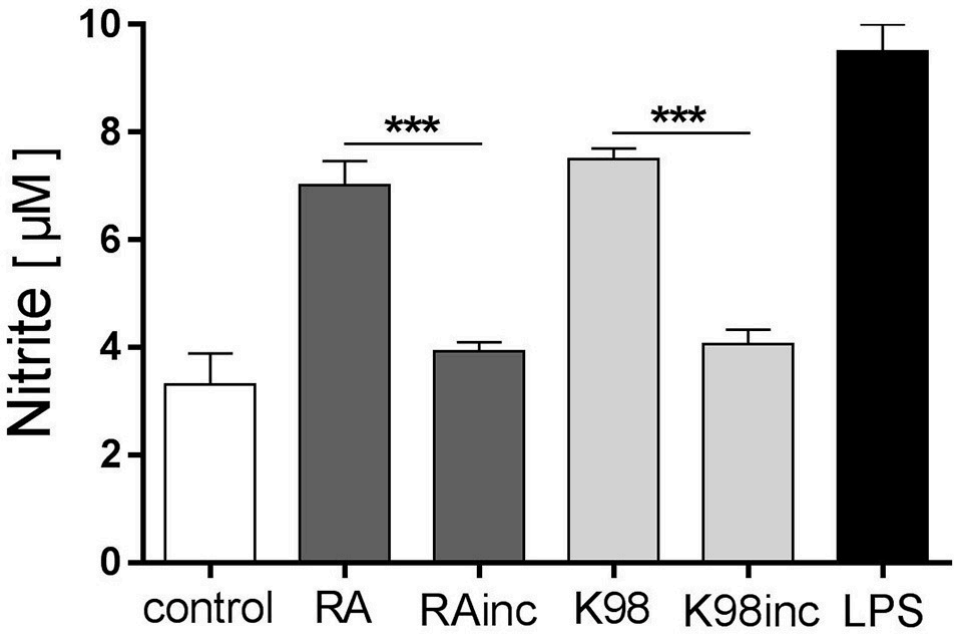
RA and K98 stimulated NO production. Macrophages were stimulated with 50 μg/ml of RA, RAinc, K98 and K98inc or 300 ng/ml LPS or 0.5% EtOH (control), in the presence of 600 pg/ml IFN-γ, for 48 h. NO levels were determined in supernatants as nitrite production using the Griess reaction. Each bar represents the mean ± SEM of triplicate determinations from three independent experiments. RA, total lipid extract from amastigotes of RA *T. cruzi* strain; RAinc, total lipid extract from incubated amastigotes of RA *T. cruzi* strain; K98, total lipid extract from amastigotes of K98 *T. cruzi* strain; K98inc: total lipid extract from incubated amastigotes of K98 *T. cruzi* strain; ^***^ statistically significant (*p* < 0.001).

Thereafter, we evaluated if all lipid extracts were able to promote NO production, a major effector molecule with trypanocidal activity, in murine peritoneal macrophages (Gutierrez et al., 2009). Results indicate that RA and K98 induced a significant NO release, whereas RAinc and K98inc did not stimulate the production of this soluble factor compared to control. Particularly, RA and K98 induced ~1.79 and 1.83-fold higher NO levels with respect to their corresponding incubated lipid extracts, RAinc and K98inc (Figure 7).

### RA and K98 induced TNF-α release

**Figure 8:**
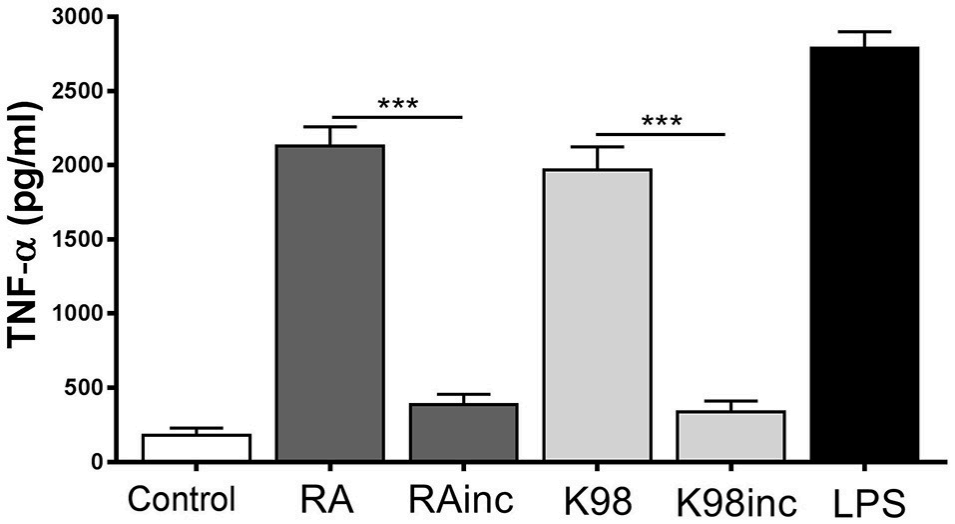
RA and K98 induced TNF-α release. Macrophages were stimulated with 50 μg/ml of RA, RAinc, K98 and K98inc or 300 ng/ml LPS or 0.5% EtOH (control), for 24 h. TNF-α levels were determined by ELISA in cell supernatants. Each bar represents the mean ± SEM of triplicate determinations from three independent experiments. RA: total lipid extract from amastigotes of RA *T. cruzi* strain; RAinc: total lipid extract from incubated amastigotes of RA *T. cruzi* strain; K98: total lipid extract from amastigotes of K98 *T. cruzi* strain; K98inc: total lipid extract from incubated amastigotes of K98 *T. cruzi* strain; ^***^ statistically significant (*p* < 0.001).

It has been reported that during the initial steps of *T. cruzi* infection a strong inflammatory response is triggered with production of cytokines, such as TNF-α, which activate cells for parasite control (Andrade et al., 2014). In view of that, we here analyzed if the lipid extracts were able to promote the production of this pro-inflammatory cytokine in murine peritoneal macrophages. Figure 8 shows that both RA and K98 induced significant levels of TNF-α with respect to control, in contrast to RAinc and K98inc which had no effect. The comparison between RAinc vs RA and K98inc vs. K98 displayed a reduction in this cytokine level of ~8.91 and 11.33 folds, respectively.

## Discussion

This is a novel report that describes the biological effect of total lipid extracts from amastigotes of two *T. cruzi* strains, which belong to different DTUs and possess polar biological behavior: RA (Tc VI, high virulence) and K98 (Tc I, low virulence) (González Cappa et al., 1980, 1981; Zingales et al., 2009). Herein, we analyzed the lipid composition of the total lipid extracts from these parasites and demonstrated that although RA and K98 had different content of phospholipids (PC and PE) and FA, they were able to induce a similar pro-inflammatory response. Remarkably, when we evaluated the effect of RAinc and K98inc, we determined the loss/decrease of the pro-inflammatory response previously detected with RA and K98, a fact that could be attributed to the quantitative changes in the FFA, phospholipid and lysophospholipid fractions. In this regard, we here demonstrated that *T. cruzi* PLA_1_, an enzyme capable of hydrolyzing zwitterionic phospholipids such as PC and PE (Wainszelbaum et al., 2001), participates in these lipid modifications. Furthermore, the quantitative decrease in lysophospholipids observed in RAinc and K98inc could be attributed to the lysophospholipase activity that *T. cruzi* PLA_1_ also possesses, since no lysophospholipase A inhibitors were used (Wainszelbaum et al., 2001; Belaunzarán et al., 2013).

Lysophospholipids, mediators in the synthetic pathways of various phospholipids and structural components, can also act as signaling molecules (Sowinska et al., 2016). A growing body of evidence suggests that LPC plays a role in the regulation of cell immune responses and chronic diseases progression (Matsumoto et al., 2007; Silva-Neto et al., 2016). Besides, it has been proposed that LPC is a dual activity molecule, able to trigger a classical pro-inflammatory phenotype as well as to induce an anti-inflammatory phenotype in macrophages (Carneiro et al., 2013; Assunção et al., 2017). As concerns *T. cruzi* LPC, this parasite synthesizes at least five species of this molecule and only sn-1 C18:1(D9)-LPC is able to promote rabbit platelet aggregation (Gazos-Lopes et al., 2014). Therefore, we could not discard the possibility that RA and K98 might possess this bioactive LPC which could be degraded by lysophospholipase activity during incubation and as consequence its content reduced in RAinc and K98inc. As regards FA, other bioactive molecules present in *T. cruzi* lipid extracts, one of the majors was palmitic acid, which can display a pro-inflammatory effect (Ajuwon and Spurlock, 2005). Stearic acid, with known anti-inflammatory effect (Pan et al., 2010; Othman Razi et al., 2015), was another major FA in *T. cruzi* lipid extracts and at variance was increased in RAinc and K98inc, a fact that could contribute to the reduction of the pro-inflammatory effects induced by RA and K98. Besides, other FAs present in low quantities as well as other phospholipids/lysophospholipids could also contribute to the global effect observed with each *T. cruzi* lipid extract.

In the present work we demonstrated, in HEK transfected cells, that the TLR2/6 heterodimer participates in the recognition of all the lipid extracts from both *T. cruzi* strains, but not TLR2/1 and TLR4. It has been described that synthetic LPC signals via TLR2 and TLR4 and the schistosomal-derived LPC via TLR2 (Magalhães et al., 2010; Carneiro et al., 2013), thus allowing us to suggest that the LPC present in the *T. cruzi* lipid extracts here studied could contribute to TLR2/6 stimulation. As regards known *T. cruzi* TLR2/6 ligands, up to now there are no reports about molecules of exclusively lipid nature, nevertheless complex glycolipids like trypomastigote mucin GPI anchors, especially the unsaturated fatty acid at the sn-2 position, have been described as potent stimulators of this heterodimer (Cardoso et al., 2016). *In vitro* stimulation of TLR2/6 by *T. cruzi* GPI-mucins leads to the production of pro-inflammatory cytokines such as IL-12 and TNF, as well as NO, which are related to a Th1-focused immune response that is important to control parasitemia and tissue parasitism (Cardoso et al., 2016). Although, *in vitro* stimulation of TLR2/6 by *T. cruzi* GPI-mucins induces pro-inflammatory cytokines release, *in vivo* assays showed that TLR2 could play an immunoregulatory role during *T. cruzi* infection (Cardoso et al., 2016). Concerning FFAs present in *T. cruzi* lipid extracts, these molecules might also contribute to the TLR2/6 stimulation here observed, since it has been described that they can activate TLR4, TLR2/1, or TLR2/6 leading to stimulation of specific signaling pathways followed by expression of TNFα, IL-6, and MCP-1 (Neacsu et al., 2013). Further studies will elucidate which particular lipid molecules, present in these lipid extracts, participate in the immunomodulation via TLR2/6. It is well known that in the TLRs pathways several transcription factors such as NF-κB, among others, are activated and induce the release of inflammatory cytokines, chemokines and NO (Shoda et al., 2001). In this concern, it has been reported that *T. cruzi* infection of murine cardiomyocytes triggers signal transduction pathways that leads to NF-κB activation, among other transcription factors (Huang et al., 2003). Herein, we determined in HEK transfected cells that all *T. cruzi* lipid extracts promoted the activation of NF-κB pathway specifically via TLR2/6 with the resulting IL-8 secretion, major chemoattractant of neutrophils. Interestingly, the fact that RAinc and K98inc induced a significantly lower NF-κB activation and IL-8 secretion with respect to RA and K98, could be attributed to the loss of some bioactive pro-inflammatory components or to the generation of compounds with anti-inflammatory effect, as previously suggested.

Macrophages are crucial cells of innate immunity that can recognize pathogens and induce LB formation, lipid mediators and cytokine release. During *T. cruzi* infection it has been reported that LB formation and COX-2 expression increase in macrophages and that LB generation occurs through a TLR2 dependent mechanism (D'Avila et al., 2011). The present finding that RA and K98 induced in macrophages the increase in LB number, together with the fact that all lipid extracts were recognized by TLR2/6 in HEK transfected cells, led us to propose that LB formation and COX-2 expression induced by *T. cruzi* lipids can also occur through a TLR2/6 dependent mechanism. Remarkably, in the case of RAinc stimulated macrophages, LB formation was significantly reduced with respect to control cells suggesting the presence in this extract of hydrolyzed lipid molecules that could inhibit the formation of these structural markers of inflammation. Besides, the findings that only RA and K98 were able to induce NO secretion and TNF-α release, pro-inflammatory mediators regulated via NF-κB, support again our point of view that the differences in the lipid composition of RAinc and K98inc here detected could be related to their reduced biological effect. On the other hand, since it has been described that macrophages possess a secreted PLA_2_, we cannot rule out that this enzyme could be up-regulated by *T. cruzi* lipids during the *in vitro* assays here described, and consequently contribute to the generation of bioactive lipids (Ruipérez et al., 2007).

Collectively, our results point to a role of *T. cruzi* lipids in the induction of a pro-inflammatory response through the TLR2/6 pathway that could contribute to the control of infection and host survival. Since up to now there is no effective vaccine against this parasite, research has focused on the identification and characterization of PAMPs from *T. cruzi* to offer new TLR agonists as possible adjuvant molecules in new generation vaccines. Further studies of *T. cruzi* lipids will elucidate which are the inmunomodulating molecules present in these extracts that could be considered as potential candidates to be tested in therapeutic approaches or immunoprophylactic strategies.

## Author contributions

EB, AC, GG, EL, PB, and MB contributed to the design of this study. EB, AC, GG, ML, GA, and MB performed the experiments. EB, AC, GG, GA, PB, and MB analyzed the data. EB, AC, GG, EL, PB, and MB wrote the manuscript.

### Conflict of interest statement

The authors declare that the research was conducted in the absence of any commercial or financial relationships that could be construed as a potential conflict of interest.

## Footnotes

^1^Available online at: http://www.who.int/mediacentre/factsheets/fs340/en/ (Accessed April 24, 2017).
