# Supplementary material for: Lipids From Trypanosoma cruzi Amastigotes of RA and K98 Strains Generate a Pro-inflammatory Response via TLR2/6
Source: Front Cell Infect Microbiol. 2018 May 8;8:151. doi: 10.3389/fcimb.2018.00151 (PMC5952039; doi:10.3389/fcimb.2018.00151)
Supplement: Supplementary file 1 [file Image_1.PDF]

Supplementary Material

Supplementary Material
Lipids from *Trypanosoma cruzi* amastigotes of RA and K98 strains generate a pro-inflammatory response via TLR2/6

Emanuel Bott, Alan Brito Carneiro, Guadalupe Gimenez, María Gabriela López, Estela María Lammel, Georgia Correa Atella, Patricia Torres Bozza and María Laura Belaunzarán*

*** Correspondence: María Laura Belaunzarán**: mbelaunzaran@fmed.uba.ar

Supplementary Figure 1

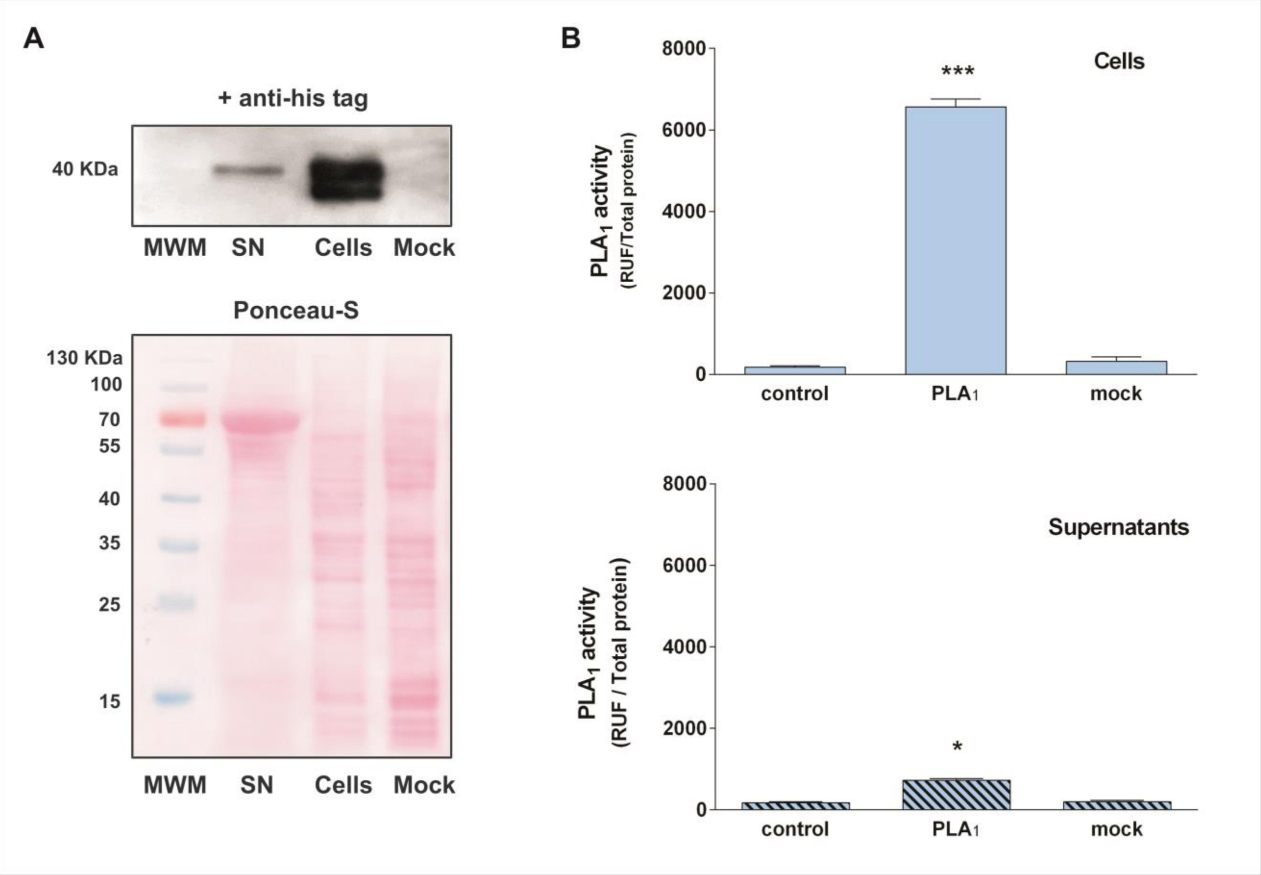

Supplementary Figure 1: For expression of recombinant *T. cruzi* PLA_1, a baculovirus-insect cell system was used. At 5 dpi, Sf9 cells infected with recombinant baculovirus-*T. cruzi* PLA_1 (cells) and their corresponding supernatants (SN), as well as control cells infected with recombinant baculoviruses of an unrelated protein (mock) were harvested. A) Samples were separated on 10%

SDS-PAGE gels, analyzed by immunoblot using a monoclonal anti-histidine specific antibody and developed by chemiluminescence. As loading control, the nitrocellulose membrane was stained with Ponceau-S. MWM: molecular weight markers. B) PLA_1 activity was determined in SF9 infected cells (PLA_1 or mock) and their corresponding supernatants using a similar protocol as described by Reisfeld et al. (1994). Aliquots of each sample were incubated with the fluorescent substrate 1-palmitoyl-2-{6-[(7-nitro-2-1,3-benzoxadiazol-4-yl)amino]hexanoyl}-sn-glycero-3-phosphocholine (NBD-PC) for 2 h at 37°C. Reaction was stopped adding 0.9 vol of 0.2M ammonia in methanol and 0.9 vol of chloroform. Fluorescence was quantified in the aqueous phase and the results are expressed as relative units of fluorescence (RUF) with respect to the total protein of each sample.
