# Supplementary material for: Lipids From Trypanosoma cruzi Amastigotes of RA and K98 Strains Generate a Pro-inflammatory Response via TLR2/6
Source: Front Cell Infect Microbiol. 2018 May 8;8:151. doi: 10.3389/fcimb.2018.00151 (PMC5952039; doi:10.3389/fcimb.2018.00151)
Supplement: Supplementary file 2 [file Image_2.PDF]

Supplementary Material

Lipids from *Trypanosoma cruzi* amastigotes of RA and K98 strains generate a pro-inflammatory response via TLR2/6

Emanuel Bott, Alan Brito Carneiro, Guadalupe Gimenez, María Gabriela López, Estela María Lammel, Georgia Correa Atella, Patricia Torres Bozza and María Laura Belaunzarán*

*** Correspondence: María Laura Belaunzarán**: mbelaunzaran@fmed.uba.ar

Supplementary Figure 2

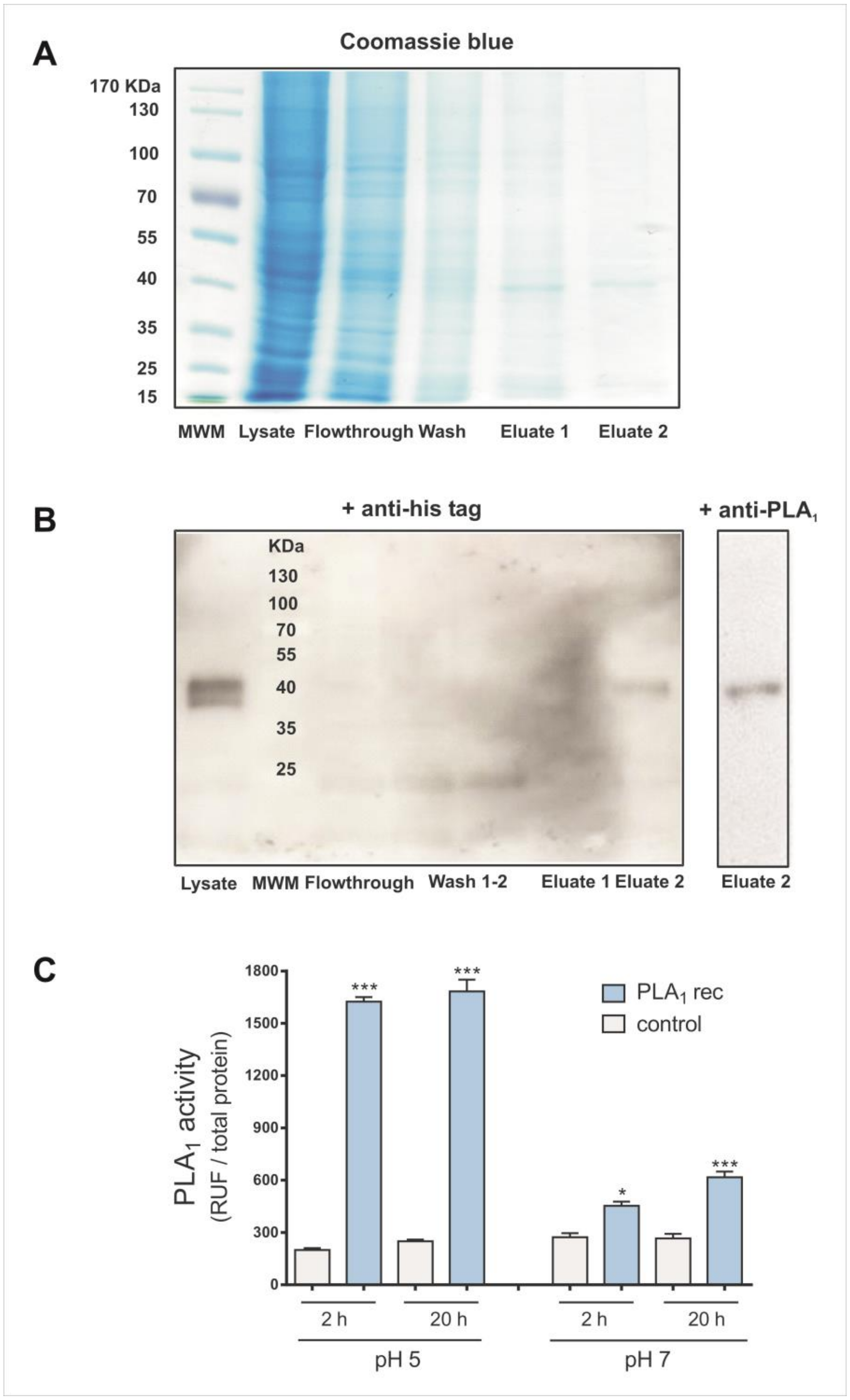

Supplementary Figure 2: Recombinant *T. cruzi* PLA_1, was purified by affinity chromatography with a Niquel-Agarose resin and aliquots of the different samples were analyzed on 10% SDS-PAGE gels by: A) Coomassie blue staining; B) immunoblot using a monoclonal anti-histidine primary antibody or anti-*T. cruzi* PLA_1 serum and developed by chemiluminescence.

C) PLA_1 activity was determined in Sf9 cells infected with recombinant baculovirus-*T. cruzi* PLA_1 using a similar protocol as described by Reisfeld et al. (1994). Aliquots of each sample were incubated with the fluorescent substrate NBD-PC for 2 h or 20 h at pH 5 or 7, at37°C. Reaction was stopped adding 0.9 vol of 0.2M ammonia in methanol and 0.9 vol of chloroform. Fluorescence was quantified in the aqueous phase and the results are expressed as relative units of fluorescence (RUF) with respect to the total protein of each sample.
